# A recurrent deletion in the SLC5A2 gene including the intron 7 branch site responsible for familial renal glucosuria

**DOI:** 10.1038/srep33920

**Published:** 2016-09-26

**Authors:** Xiangzhong Zhao, Li Cui, Yanhua Lang, Ting liu, Jingru Lu, Cui Wang, Sylvie Tuffery-Giraud, Irene Bottillo, Xinsheng Wang, Leping Shao

**Affiliations:** 1 Central Laboratory, The Affiliated Hospital of Qingdao University, 1677 Wutaishan Road, Qingdao 266555, China; 2 Department of Nephrology, the Affiliated Hospital of Qingdao University, 16 Jiangsu Road, Qingdao 266003, China.; 3 Laboratory of Genetics of Rare Diseases, EA7402, University of Montpellier, F-34000, France; 4 Division of Medical Genetics, Department of Molecular Medicine, Sapienza University, San Camillo-Forlanini Hospital, Circ. Gianicolense, 87, Padiglione Morgagni 00152, Rome, Italy; 5 Urology, Affiliated Hospital, Qingdao University, Qingdao 266003, China

## Abstract

Familial renal glycosuria (FRG) is caused by mutations in the SLC5A2 gene, which codes for Na^+^-glucose co-transporters 2 (SGLT2). The aim of this study was to analyze and identify the mutations in 16 patients from 8 families with FRG. All coding regions, including intron-exon boundaries, were analyzed using PCR followed by direct sequence analysis. Six mutations in SLC5A2 gene were identified, including five missense mutations (c.393G > C, p.K131N; c.1003A > G, p.S335G; c.1343A > G, p.Q448R; c.1420G > C, p.A474P; c.1739G > A, p.G580D) and a 22-bp deletion in intron 7 (c.886(-10_-31)del) removing the putative branch point sequence. By the minigene studies using the pSPL3 plasmids, we confirmed that the deletion c.886(-10_-31)del acts as a splicing mutation. Furthermore, we found that this deletion causes exclusion of exon 8 in the SCL5A2 transcript in patients. The mutation c.886(-10_-31)del was present in 5 (62.5%) of 8 families, and accounts for about 37.5% of the total alleles (6/16). In conclusion, six mutations resulting in FRG were found, and the c.886(-10_-31)del may be the high frequency mutation that can be screened in FRG patients with uniallelic or negative SLC5A2 mutations.

The kidney contributes to glucose homeostasis by reabsorbing approximately 180 g from the glomerular filtrate each day. The occurrence of glucosuria in the absence of both generalized proximal tubular dysfunction and abnormal glucose metabolism is known as renal glucosuria and recognized as an inherited disorder and hence the designation of familial renal glucosuria (FRG). Glucosuria in these patients can range from <1 to >150 g/1.73 m^2^ per day (normal value: range 0.03 to 0.3 g/d)^1^,^2^.

At least two sodium-coupled glucose transporters, SGLT1 and SGLT2, play an important role in the apical membrane of proximal tubular cells in the kidney^3^. SGLT2 is expressed exclusively near the early proximal convoluted tubule (termed S1), whereas SGLT1 is expressed near the medullary proximal tubule (termed S3). SGLT2 is a low-affinity, high-capacity glucose transporter, whereas SGLT1 is a high-affinity, low-capacity glucose transporter. In the early proximal tubule, SGLT2 driven by the electrochemical Na^+^ gradient generated by the Na^+^/K^+^-ATPase, with Na^+^-to-glucose coupling ratio of 1:1, reabsorbs the bull of the filtered glucose^4^. Thus, the bulk of glucose is reabsorbed at the S1 segment by the high-capacity SGLT2 transporter, whereas the remaining glucose that enters the S3 segment is reabsorbed by the high-affinity SGLT1 transporter; together they minimize glucose loss in the urine.

To date, SLC5A2 (OMIM: 182381) is the only gene that has been associated to FGR. Human SLC5A2 gene has been localized to p11.2 on chromosome 16; it consists of 14 separate exons spanning approximately 7.7 kb of genomic DNA, and encodes the 672 amino acid protein SGLT2^5^.

So far, more than seventy mutations in the SLC5A2 gene have been identified to be responsible for the vast majority of cases of FRG^6^,^7^, including missense mutations, nonsense mutations, small deletions and splicing mutations, and most of the reported mutations are missense^7^,^8^,^9^,^10^,^11^,^12^,^13^,^14^,^15^,^16^,^17^,^18^,^19^. FRG is inherited as a co-dominant trait, and the majority of reported mutations are restricted to a single individual or family. Whereas the intron 7+5G > A (c.885+5G > A), meanwhile reported in several unrelated pedigrees of different ethnic origins, might be a mutational hot spot^7^,^12^. This paper expanded the study of mutation analysis of SLC5A2 gene in 16 Chinese patients from 8 families, and identified another high-frequency deletion in intron 7 resulting in abnormal splicing of exon 8 in SLC5A2 gene.

## Results

### Patients and phenotype

The clinical characterization and laboratory findings of eight probands are shown in Table 1. Among of them, five patients presented with mild glycosuria (Quantitative test for 24-hour urine glucose: 1.77–2.04 g/1.73 m^2^), two patients manifested middle degree glycosuria (Quantitative test for 24-hour urine glucose: 10.56 and 12.74 g/1.73 m^2^ respectively), and the other one patient showed severe glycosuria (Quantitative test for 24-hour urine glucose: 50.68 g/1.73 m^2^). The patient with severe glycosuria showed polyuria (approximately 3,500 ml/day) and polydipsia. One of the patients with middle degree glycosuria occasionally appeared similar symptoms of hypoglycaemic reaction, such as sweating, palpitation and sense of hunger. Besides these probands, all the other patients presented mild glycosuria, and had no obvious discomfort. The 24-hour urine glucose levels of all patients and their family members are shown in Table 2.

### Mutation Analysis in Patients with Renal Glucosuria

**Figure 1:**
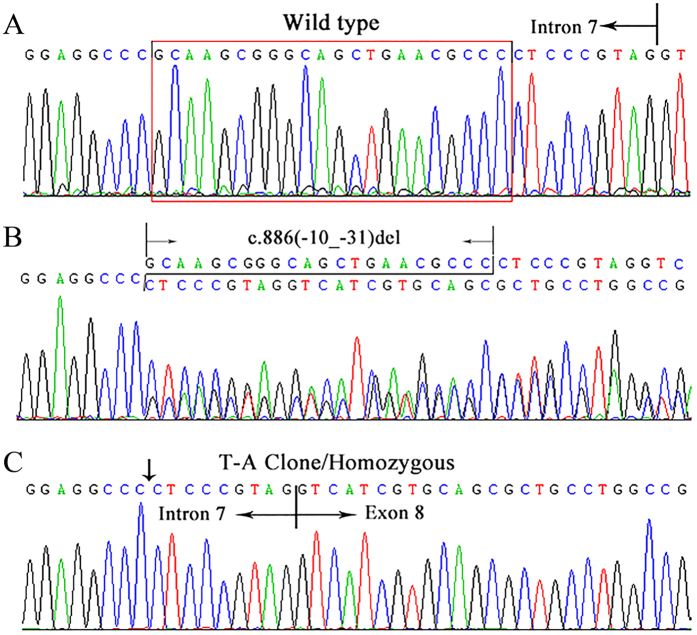
The deletion c.886(-10_-31)del in intron 7 of SLC5A2 gene identified in Chinese patients with Familial Renal Glucosuria. (**A**) Wild type electropherogram; (**B**) Electropherogram showing the mutations in the patient IVc. (**C**) Electropherogram of the T-A clone for the mutant allele. The arrow indicated the position of deletion of intron 7 in SLC5A2.

By direct sequencing analysis, six mutations in SLC5A2 were identified in eight families with FRG, including five missense variants (c.393G > C, p.K131N; c.1003A > G, p.S335G; c.1343A > G, p.Q448R; c.1420G > C p.A474P; c.1739G > A, p.G580D) (Supplemental Fig. 1), and a 22-bp deletion in intron 7 (c.886(-10_-31)delGCAAGCGGGCAGCTGAACGCCC) (Fig. 1, Table 2). The zygosity of each identified DNA variant is also reported in Table 2. Direct sequencing analysis failed to find above-mentioned mutations in 100 unrelated healthy subjects.

The data from *in silico* analysis by three different software programs SIFT, PolyPhen-2 and Mutation Taster showed that the mutations p.K131N, p.S335G, p.Q448R and p.A474P may be deleterious, and might be involved in FRG, however the variant p.G580D may be “benign” (Supplemental Table 1). The p.K131, p.S335, p.Q448 and p.A474 are highly conserved among 8 different species (human, rat, mouse, frog, zebrafish, bull, macaque and boar), however the p.G580 is conserved among only 6 species (human, rat, mouse, zebrafish, macaque and boar). Additionally, both p.K131 and p.S335 are highly conserved in the SGLTs protein families including sodium-coupled glucose transporters SGLT1 and SGLT2, glucose sensor SGLT3, SGLT4 (multi-pass membrane protein that participates in the sodium-dependent transport of D-fructose, D-mannose and Dglucose) and SGLT5. p.Q448 and p.A474 are conserved in those three and four ones respectively, while the amino acid at position 580 presents diversity however.

The identified five missense mutations are private, while the deletion c.886(-10_-31)del was found in 5 out of the 8 families. The mutation c.886(-10_-31)del accounts indeed for about 37.5% (6/16) of the total alleles. Since this mutation is located in the upstream vicinity of the 3′-splice site, it was predicted to break the putative branch point sequence causing a negative effect on the recognition of the acceptor splicing site of intron 7 by the following softwares: BDGP (the score decreases from 0.88 to 0.60 when passing from the wt to the mutant allele), NetGene2 (confidence decreases from 0.95 to 0.33) and Spliceview (score decreases from 83 to 79). Finally, this deletion was predicted to alter the splicing mechanism eliminating or creating branch point motif by the HSF3.0 bioinformatic tool (Supplemental Table 2).

**Figure 2:**
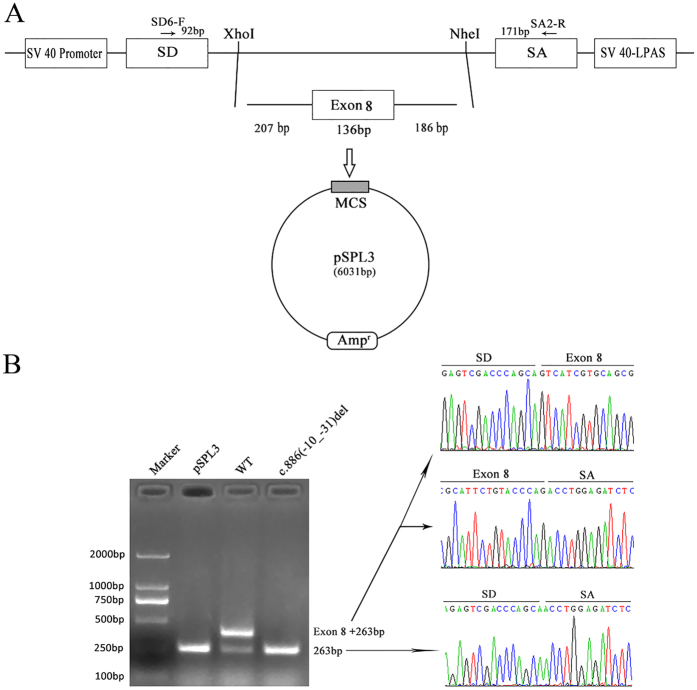
The minigene splicing assay based on the pSPL3 exon trapping vector. (**A**) The pSPL3 vector contains two exons SD and SA, and a functional intron, with transcription beginning following the SV40 promoter and ending at the LPAS (late poly (**A**) signal). Wild pSPL3-W and mutant pSPL3-M plasmids containing 207 bp of intron 7, 136 bp of exon 7 and 186 bp of intron 8 were separately cloned into the XhoI and NheI cloning sites of the pSPL3 vector. (**B**) Agarose gel electrophoresis of RT-PCR products. SD6 and SA2 primers were designed for RT-PCR amplification of cDNA sequences generated by transfected 293 T cells. Lane1: Marker; Lane2: empty vector (263 bp); Lane3: 399 bp (263 bp + 136 bp) and 263 bp; Lane 4: 263 bp. MCS: multiple cloning sites.

To define the transcript level effects of this novel deletion, we performed exon trapping using the pSPL3 plasmids. The fragments with the wild or mutant alleles involving exon 8 (136bp) flanked by upstream intronic sequence (207bp) and downstream intronic sequence (186bp), were cloned into the splicing vector pSPL3 using specific primers (Forward, 5′-CGCCTTCCCCACAACGGTCTAA-3′; Reward, 5′-CGTTAGGACGGGGCCTGGTCT-3′) linking the XhoI and NheI restriction enzyme sites (Fig. 2A), as described in our previous study^20^. The minigene assays showed that both the empty pSPL3 control and the c.886(-10_-31)del mutant constructs gave rise to a 263bp PCR fragment missing exon 8 of SLC5A2 gene, while the wild-type yielded a main RT-PCR product of 399bp containing exon 8, and a minor product of 263bp lacking exon 8 (Fig. 2B). Therefore, we determined via a combination of *in silico* and *in vitro* assays that the deletion including the putative intron 7 branch site caused exon 8 skipping in the SLC5A2 transcripts.

**Figure 3:**
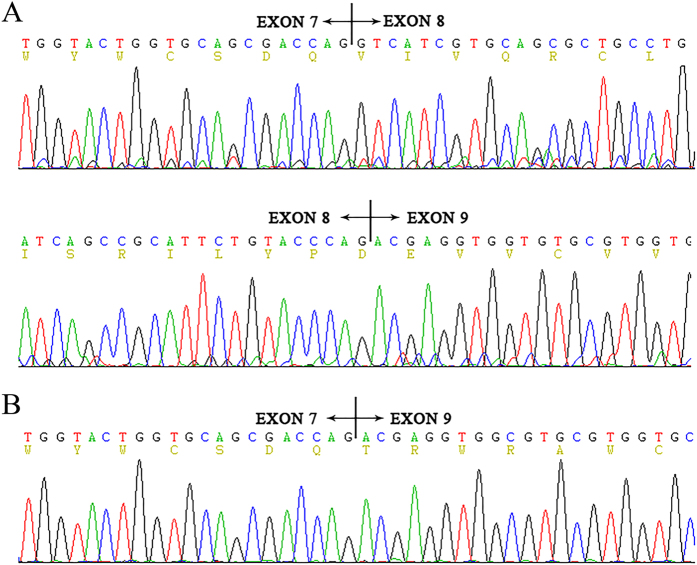
Partial RNA sequence of SLC5A2 in the patients with Familial Renal Glucosuria and normal person. (**A**) wild type electropherograms. (**B**) patient IVc electropherogram showing the absence of exon 8.

The complete skipping of exon 8 results in a 45 amino acid deletion (residues 296–340) with a subsequent frame-shift from codon 341 and premature termination at position 371 in exon 9. We then investigated whether the deletion c.886(-10_-31)del really led to exon 8 skipping in an actual patient. The cDNA from the peripheral blood was amplified by nested PCR with primers spanning exon 7 to exon 9. By cDNA sequencing, the exon 8-excluded transcript was identified only in the patient and not in a not-mutated individual (Fig. 3).

### Correlation of genotype and phenotype

As shown in Table 2, the homozygotes or compound heterozygotes cases with two mutations in SLC5A2 gene presented with middle to severe degree glucosuria (24 h glucose excretion 10.56–50.68 g/1.73 m^2^); However the carriers of heterozygous variants manifested with normal or mild glucosuria (24 h glucose excretion ≤2.45 g/1.73 m^2^). These results are in accordance with recent reports in the literature^8^,^9^ and the genotype-phenotype associations in these families fit the co-dominant inheritance pattern with variable expressivity.

The c.886(-10_-31)del heterozygous cases had normal or mild glucosuria, while the homozygous have severe glucosuria with the only exception of family V. In which the proband who carry the heterozygous c.886(-10_-31)del had middle degree glucose wasting (24 h glucose excretion 12.74 g/1.73 m^2^). To confirm whether missense mutations modulate the phenotype in the same way of the splicing mutations of c.886(-10_-31)del, we performed the comparison of 24 h glucose excretion between the cases harboring heterozygous missense variants and those harboring the heterozygous splicing mutation (excluding the family V and the case IVb without specific value). The results demonstrated that c.886(-10_-31)del heterozygotes had more severe glucosuria (Student’s t test, p = 0.04).

Additionally, three heterozygous alterations were found in family I, p.S335G came from one allele, while both p.Q448R and p.G580D from the other allele.

## Discussion

In this investigation, by mutation analysis of SLC5A2 gene in sixteen patients from eight families, we found six mutations, including four novel missense substitutions (p.K131N, p.S335G, p.Q448R and p.G580D) and one previously reported (p.A474P)^19^, as well as one novel deletion in intron 7. Analysis *in silico* programs revealed that the p.K131N, p.S335G, p.A474P and p.Q448R mutations may be pathogenic, while the p.G580D might be benign. SGLT2 have 14 transmembrane helices (TMHs) with both the hydrophobic N- and C- terminal domains lying extracellular^2^. Both p.K131 and p.G580 are situated in the cytoplasmic regions (between TMH 3 and TMH 4, TMH 13 and 14 respectively); while both p.S335 and p.Q448 are localized in the extracellular loops (between TMH 8 and TMH 9, TMH 10 and TMH 11 respectively); however p.A474 is positioned at 11^th^ TMH (Supplemental Fig. 2). The 3D SWISSMODEL and the positions of these mutations p.K131, p.S335, p.Q448 and p.A474 in it are shown in Supplemental Fig. 3. These four probably pathogenic missense mutations may lead to a reduced or abolished transporter activity by the following molecular mechanisms: (1) impaired protein synthesis, (2) impaired protein processing, (3) impaired protein insertion into the plasma membrane, (4) impaired intrinsic transporter activity, (5) accelerated protein removal or degradation, or (6) altered functional regulation. In addition, the hypothesis based on the studies in transport mechanisms of SGLT1 and/or SGLT1/SGLT3 chimeras suggested that the COOH-terminal domain (residues 407–662 containing TMHs 10–14) determined sugar affinity and selectivity, while the NH2-terminal half of the protein was involved in Na^+^ binding and coupling^21^. Therefore, the variants p.K131N and p.S335G may disturb Na^+^ binding and transport, while p.Q448R and p.A474P might interfere with glucose affinity and translocation. Of note, a recent *in vitro* functional expressing study by Yu L. *et al*. demonstrated that p.A474P probably not only alter protein processing and impair protein insertion into the plasma membrane, but also impair protein synthesis or accelerate protein removal or degradation^19^.

One of the main finding in this investigation was the identification of a new mutation, consisting of a 22-bp deletion in intron 7 (c.886(-10_-31)del) causing FRG. The deletion may destroy the putative branch point sequence located at the close upstream of the intron 7/exon 8 junction. From the results based on the minigene splicing assay and the RNA analysis from peripheral blood leucocytes, skipping of the whole exon 8 caused by this deletion will lead to a truncated protein lacking the COOH-terminal domain from TMH 8 to TMH 14, and a loss of transport activity of SGLT2. Unfortunately, we could not confirm these results on the patients’ RNA from kidney tissue. Since different types and levels of alternative tandem splice site exons occurring in different human organ systems and cell types, we cannot exclude the possibility of multiple exons skipping under the circumstance of the losing of branch site sequence of intron 7 resulted by this deletion and relative small size of adjacent introns and exons. Remarkably, the deletion c.886(-10_-31)del was the second high-frequency mutation following the intron7+5G > A (c.885+5G > A) that has been identified so far^7^,^12^. Further investigation based on more FRG patients is needed to establish whether it is a mutational hot-spot.

In keeping with the recent reports, we found that FRG inherited as a co-dominant entity with different penetrance^8^. However, the proband in family V with distinct different glucosuria from other heterozygotes harboring the same mutation c.886(-10_-31)del might carry the second mutation on the other allele which may be present in non-coding sequences. Additionally, that some other transporters, such as other SGLTs that are known to be expressed in the kidney and whose functions have not yet been clarified, may reabsorb a significant amount of glucose in these patients under certain circumstances might also explain the discrepancy to some extent. In addition, we demonstrated that c.886(-10_-31)del may be associated with a more severe phenotype than missense mutations. This may be related to that missense mutations commonly had some residual glucose reabsorption activity, while the mutation c.886(-10_-31)del resulted in the whole abnormally spliced mRNA as shown in Fig. 2.

In summary, we identified six mutations, including five missense variants and a 22-bp deletion in intron 7 with high mutation rate, in eight Chinese families with FRG. Furthermore, we confirmed that this deletion was a splice pathogenic mutation resulting in exon 8 skipping probably through eliminating the branch site sequence. Such data might assist in more rapid diagnosis or screening this hereditary disease FRG.

## Subjects and Methods

### Diagnostic Criteria

There are different diagnostic criteria in different historical stages. We use the following diagnostic criteria: (1) 24-hour urine glucose >0.3 g/1.73 m^2^; (2) Normal glucose metabolism; (3) No evidence of other kidney disease, such as hematuria, proteinuria, acidaminuria, or phosphaturia, and normal renal function; (4) Except for pregnant women. Mild glucosuria, 24-hour urine glucose <10.0 g/1.73 m^2^; medium glucosuria, 10.0 g/1.73 m^2^ ≤24-hour urine glucose <20.0 g/1.73 m^2^; severe glucosuria ≥20.0 g/1.73 m^2^.

### Patients

The study protocol was approved by the Ethics Committee of the Affiliated Hospital of Qingdao University and the methods were carried out in accordance with the approved guidelines. Informed consent was obtained from each subject. The study group consisted of 16 patients belonging to 8 unrelated families including total 26 family members. The mean age of these patients was 36.6 years (22–54 years).

#### Mutation Analysis

Genomic DNA was extracted from the peripheral blood of the patients and their family members by GenElute blood genomic DNA kit (Sigma, NA2010). Fourteen pairs of oligonucleotide primers were generated to amplify all exons and flanking intronic regions of the SGLT2 gene. PCRs were performed in 25 μl of solution containing 0.2 mM dNTP, 0.03 U/μl Taq polymerase (Takara EX Taq Hot start version, DRR006B), 2.0 mM MgCl_2_, 2.5 μl 10 × PCR Mg^2+^-free Buffer (Takara), approximately 30 ng genomic DNA, and 1 mM of each primer. Gradient PCRs were performed with an initial denaturation step at 95 °C for 5 min subsequently followed by 33 cycles with denaturation at 95 °C for 45 s, annealing at 58–64 °C for 45 s, and elongation at 72 °C for 45 s. PCR samples were subjected to bidirectional sequencing. The sequence reactions were run on an ABI Prism 3700 DNA Analyzer (Applied Biosystems). When heterozygous deletion or insertion mutations were suspected by direct sequence data, the PCR products were subcloned into PGEM-T Easy vectors with a cloning kit (Promega, A1360) and then sequenced with T7/SP6 sequencing primers. Amino acid substitutions not previously reported were evaluated using the in silico prediction programs SIFT, PolyPhen-2 and Mutation Taster. On the other hand, for amino acid substitutions, multiple sequence alignments using SGLT2 orthologs of rat (Rattus norvegicus, NP_072112.2), mouse (Mus musculus, NP_573517.1), frog (Xenopus Laevis, NP_00108799.1), zebrafish (Danio rerio, AAH67629.1), bull (Bos Taurus, NP_97623.1), macaque (Macaca malatta, XP_001113206.1), boar (Suc scrofa, XP_005662085.1) were used to evaluate evolutionary conservation. Multiple sequence alignments were also performed among the SGLTs family members including SGLT1 isoform 1 (NP_000334.1), SGLT2 (NP_003032.1), SGLT3 (NP_055042.1), SGLT4(NP_001011547.2) and SGLT5 isoform 1 (NP_689564.3).

To analyze the potential effect of variants (atypical splice site mutations) in the splice prediction, in silico analyses were performed. Four programs were used: BDGP (available at http://www.fruitfly.org), HSF 3.0 (available at http://www.umd.be/HSF3/HSF.html), NetGene2 (available at http://www.cbs.dtu.dk/services/NetGene2/), and Spliceview (available at http://bioinfo4.itb.cnr.it/~webgene/wwwspliceview_ex.html).

#### Minigene Constructions and Expression

To confirm the probable splice mutation, *in vitro* analysis was performed using a minigene splicing assay based on the pSPL3 exon trapping vector^20^,^22^. Fragments with the wild or mutant alleles containing exon of interest, flanked by upstream intronic sequence and downstream intronic sequence, were cloned into the splicing vector pSPL3 using specific primers linking the XhoI and NheI restriction enzyme sites (TGGAGC^TCGAG: XhoI; AATTTG^CTAGC: NheI). The ancestral and mutant type constructs were named pSPL3-W and pSPL3-M, respectively. All constructs were verified to contain the correct sequence by direct sequencing.

Human epithelial kidney 293 T (HEK 293 T) cells were cultured in DMEM medium containing 10% fetal bovine serum (FBS), penicillin (100 U/L), and streptomycin (100 mg/L) at 37 °C in a 5% CO_2_ atmosphere. One day before transfection, cells were transferred to 6-well culture plate to grow to approximately 70% to 80% confluence in an antibiotic free medium. Cells were then transfected with 4 μg plasmid DNA (pSPL3-W, pSPL3-M and empty pSPL3-control each) using OPTI-MEM^®^ IMedium and Lipofectamine 2000 (Invitrogen, Carlsbad, CA, USA) according to the manufacturer’s instructions. Cells were harvested, total RNA was extracted after 24 h transfection with the RNAsimple Total RNA Kit (Tiangen, Beijing, China) and used for RT-PCR to confirm the splicing patterns. First strand cDNA was synthesized from 2 to 3 μg of total RNA by random-primed reverse transcription with Superscript II Reverse Transcriptase (Invitrogen Corporation, Carlsbad, CA). To evaluate the pattern of transcripts from the transfected minigenes, the following vector-specific primers were used for RT-PCR amplification: a forward primer SD6 (5′-TCTGAGTCACCTGGACAACC-3′) and a reverse primer SA2 (5′-ATCTCAGTGGTATTTGTGAGC-3′). The PCR amplification reaction was performed as follows: in 50 μL volume, 2 μl of cDNA, 5 μl of Expand High Fidelity buffer 3 (Roche, Mannheim, Germany), 1 μM of each primer, 0.8 μM dNTPs, and 2.6 U Expand High Fidelity enzyme mix (Roche, Mannheim, Germany) in a 9700 (Applied Biosystem, FosterCity, CA, USA) thermal cycler. Thermal conditions were 30 cycles of 95 °C for 30 seconds, 58 °C for 30 seconds, and 68 °C for 1 minute, preceded by 2 minutes at 95 °C, and followed by a final elongation step at 68 °C for 10 minutes. The PCR products were separated by electrophoresis on a 3% agarose gel and each band signal was quantified by Quantity One software (Bio-Rad, Richmond, CA). All transcripts were analyzed by sequencing.

#### RNA analysis

cDNA was reverse transcribed from total RNA extracted from peripheral blood leucocytes. Splice mutations were detected by cDNA-sequencing using nested PCR primers. The primers used for the 1^st^ PCR analysis were 5′-TTCACCAAGATCTCAGTGGA-3′ and 5′-CCTGGGGCTCATTCATCT-3′, those for 2^nd^ were 5′-ATGGGTTACGCCTTCCACGAG-3′ and 5′-CAGACCGTTGGGCATGAGCT-3′.

## Additional Information

**How to cite this article**: Zhao, X. *et al*. A recurrent deletion in the SLC5A2 gene including the intron 7 branch site responsible for familial renal glucosuria. *Sci. Rep.* **6**, 33920; doi: 10.1038/srep33920 (2016).

## Supplementary Material

Supplementary Information

## Figures and Tables

**Table 1:** Clinical characteristics of seven probands with familial renal glucosuria.

Clinical characteristics	Ic	IIc	IIIc	IVc	Vc	VIa	VIIc	VIIIa
Age (years)	22	26	30	32	25	52	38	48
Gender	Male	Male	Male	Female	Male	Male	Male	Female
Height (cm)	178	175	176	168	175	172	170	162
Weight (Kg)	62	66	78	60	72	75	63	62
FPG (mmol/l)^a^	4.38	4.52	5.42	4.19	5.29	4.89	4.01	4.79
2hPBG (mmol/l)^b^	5.98	6.07	6.24	4.8	6.02	6.33	6.17	5.99
HbA1c (%)	4.2	4.8	5.0	4.1	5.4	4.7	4.7	4.9
SCr (μmol/l)	100.2	86.3	93.2	71.0	83.5	82.3	83.2	69.0
eGFR (ml/min/1.73 m^2^)^c^	89.25	106.58	112.54	97.1	111.6	94.0	102.3	90.1
CHOL (mmol/l)	2.86	3.92	5.16	3.15	3.32	4.77	4.22	4.66
TG (mmol/l)	0.32	0.49	0.92	0.41	0.77	1.42	0.77	1.33
Uric Acid (μmol/l)	97.8	151.1	256.3	172.3	325	398	299.3	194.6
FUG (mmol/l)^d^	7.46	0.53	0.25	0.62	3.02	0.42	14.55	1.44
PUG (mmol/l)^e^	44.27	22.03	22.71	18.77	60.39	16.42	105.4	19.55
24 h Urine Glucose (g/24 h/1.73 m^2^)	10.56	1.96	1.77	1.66	12.74	1.34	50.68	1.78
Urine pH	6.5	8.0	6.0	5.5	5.5	6.0	6.0	6.0
Urine gravity	1.020	1.015	1.022	1.021	1.025	1.018	1.028	1.025
Proteinuria	—	—	—	—	—	—	—	—
Aminoaciduria	—	—	—	—	—	—	—	—

^a^FPG, fasting plasma glucose;

^b^2hPBG, 2-hours postprandial blood glucose;

^c^eGFR, calculated by CKD-EPI formula;

^d^FUG, Fasting urine glucose;

^e^PUG, postprandial urine glucose

**Table 2:** Results of glucose excretion studies and mutation analysis in patients with familial renal glucosuria and their family members.

Family-member	24 h Glucose excretion (g/24 h/1.73 m^2^)	Allele 1	Allele 2
**Ic (proband)**	**10.56**	**p.S335G**	**p.Q448R+ p.G580D**
Ia (father)	0.15	p.S335G	WT
Ib (mother)	0.10	WT	p.Q448R+ p.G580D
**IIc (index case)**	**1.96**	**c.886(-10_-31)del**	**WT**
**IIa (father)**	**1.88**	**c.886(-10_-31)del**	**WT**
IIb (mother)	0.08	WT	WT
**IIIc (proband)**	**1.77**	**c.886(-10_-31)del**	**WT**
**IIIa (father)**	**2.04**	**c.886(-10_-31)del**	**WT**
IIIb (mother)	2.3^a^	WT	WT
IIId (brother)	0.01	WT	WT
IIIe (daughter)	0.02	WT	WT
**IVc (proband)**	**1.66**	**c.886(-10_-31)del**	**WT**
IVa (father)	Φ	WT	WT
IVb (mother)	Φ	c.886(-10_-31)del	WT
**Vc (proband)**	**12.74**	**c.886(-10_-31)del**	**WT**
Va (father)	0.03	WT	WT
**Vb (mother)**	**0.88**	**c.886(-10_-31)del**	**WT**
**Via (proband)**	**1.34**	**p.A474P**	**WT**
**VIb (son)**	**2.25**	**p.A474P**	**WT**
**VIIc (proband)**	**50.68**	**c.886(-10_-31)del**	**c.886(-10_-31)del**
**VIIa (father)**	**1.44**	**c.886(-10_-31)del**	**WT**
**VIIb (mother)**	**2.45**	**WT**	**c.886(-10_-31)del**
**VIId (daughter)**	**1.78**	**c.886(-10_-31)del**	**WT**
**VIIIa (proband)**	**1.58**	**p.K131N**	**WT**
VIIIb (spouse)	0.17	WT	WT
**VIIIc (son)**	**1.37**	**p.K131N**	**WT**

Patients with familial renal glucosuria are indicated by boldface; wt, wild type; Ø, no glucosuria by dipstick method;

^a^at elevated blood glucose concentrations as a result of type 2 diabetes.
