# Supplementary material for: A recurrent deletion in the SLC5A2 gene including the intron 7 branch site responsible for familial renal glucosuria
Source: Sci Rep. 2016 Sep 26;6:33920. doi: 10.1038/srep33920 (PMC5036194; doi:10.1038/srep33920)
Supplement: Supplementary Information [file srep33920-s1.pdf]

Title: **A recurrent deletion in the SLC5A2 gene including the intron 7 branch site responsible for familial renal glucosuria**

Authors: Xiangzhong Zhao[a], Li Cui[b], Yanhua Lang[b], Ting liu[a,b], Jingru Lu[a,b], Cui Wang[a,b], Sylvie Tuffery-Giraud[c], Irene Bottillo[d], Xinsheng Wang[e], Leping Shao[a,b]

[a]Central Laboratory, [b]Department of Nephrology and [e]Urology, Affiliated Hospital, Qingdao University, Qingdao 266003, China; [c]Laboratoire de Génétique de Maladies Rares, Université de Montpellier, France; [d]Division of Medical Genetics, Department of Molecular Medicine, Sapienza University, San Camillo-Forlanini Hospital, Rome, Italy.

Corresponding Author:

Leping Shao, Central Laboratory and Department of Nephrology, the Affiliated Hospital of Qingdao University, 16 Jiangsu Road, Qingdao 266003, China. Tel: +8653282911301; Fax: +8653282911093; E-mail: lepingshao@163.com

And

Xinsheng Wang, Department of Urology, the Affiliated Hospital of Qingdao University, 16 Jiangsu Road, Qingdao 266003, China. Tel: +8653282911801; Fax: +8653282911188; E-mail: qy_wangxs@126.com

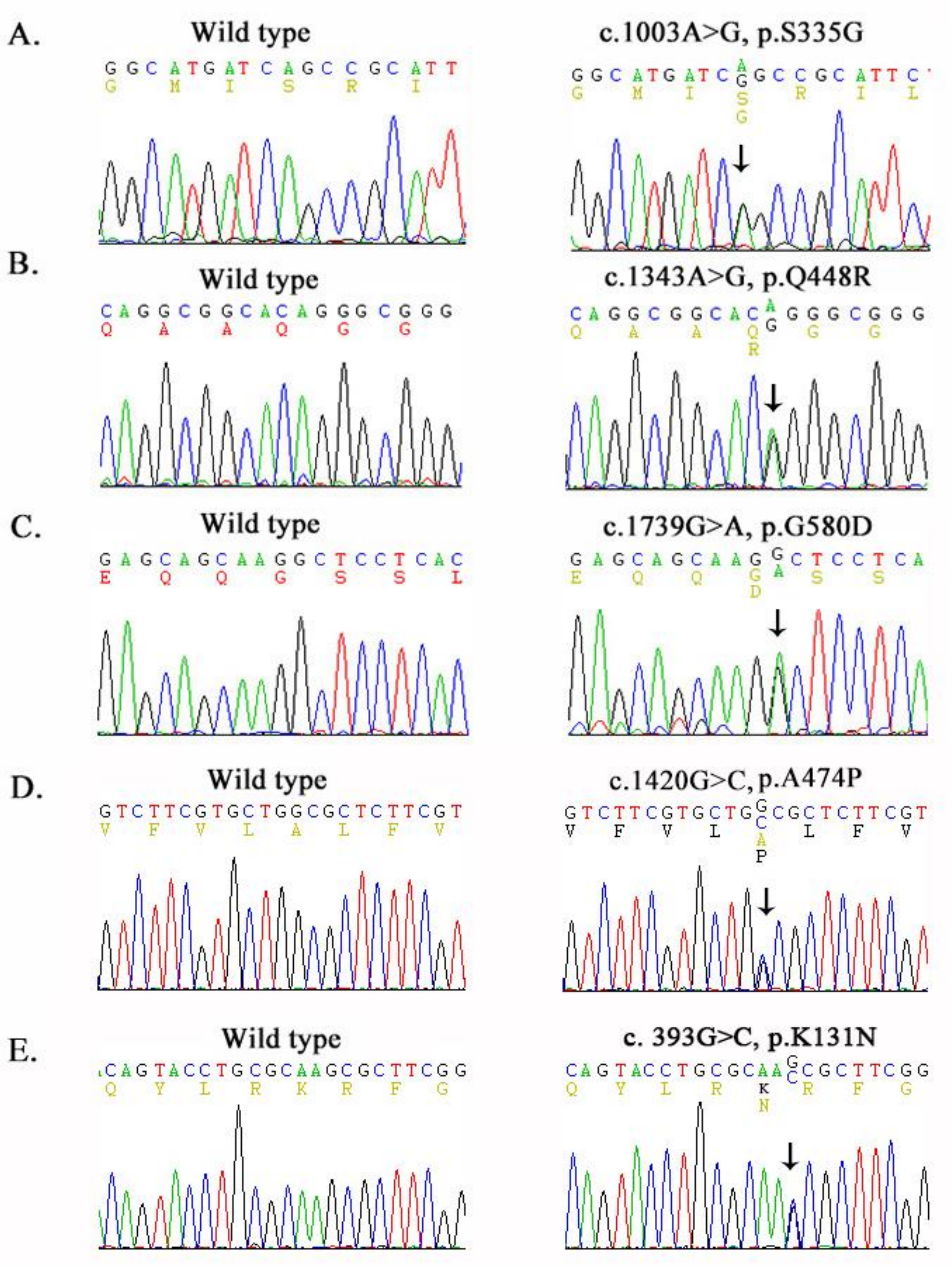

Supplemental Figure 1. SLC5A2 missense mutations identified in Chinese patients with Familial Renal Glucosuria.

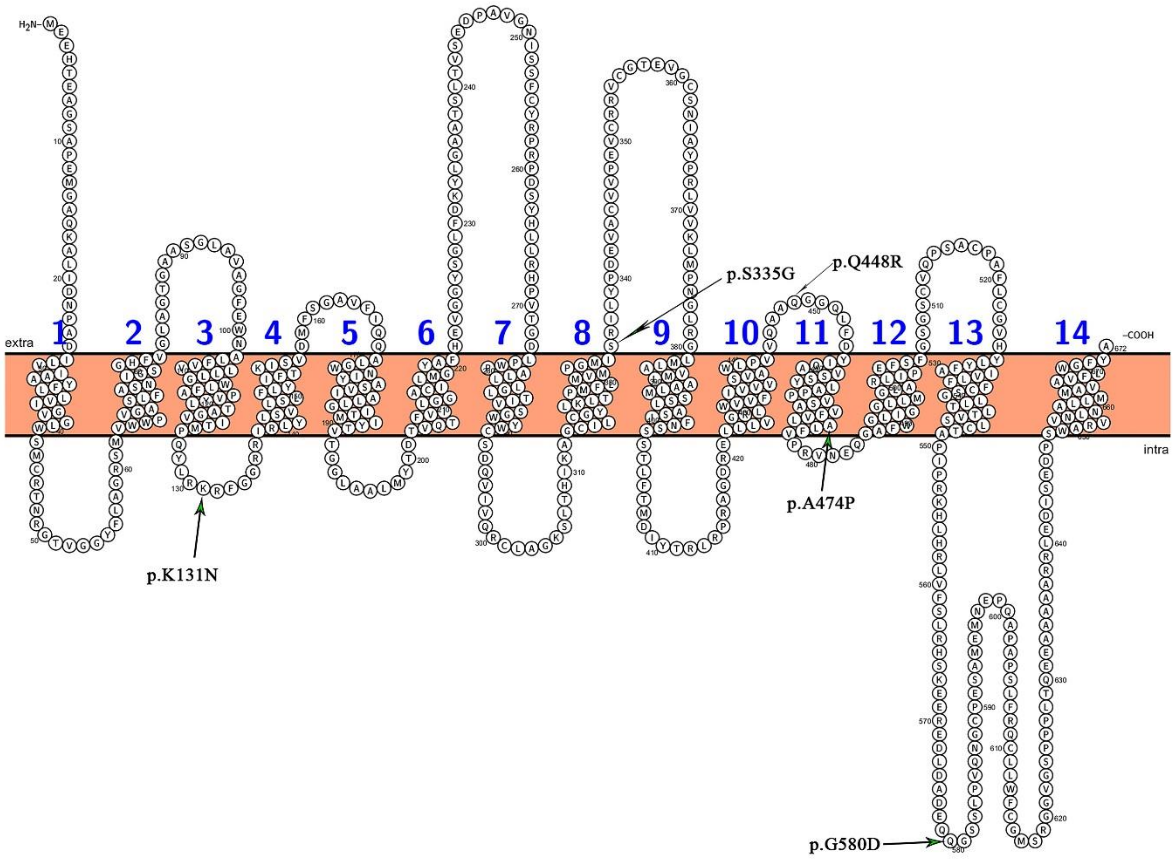

Supplemental Figure 2. Schematic diagram of the SGLT2 and missense mutations in Chinese Familial Renal Glucosuria patients. The SGLT2 is represented as a 14-transmembrane-domain protein with extracellular amino and carboxyl termini. The sites of mutations are denoted by arrows.

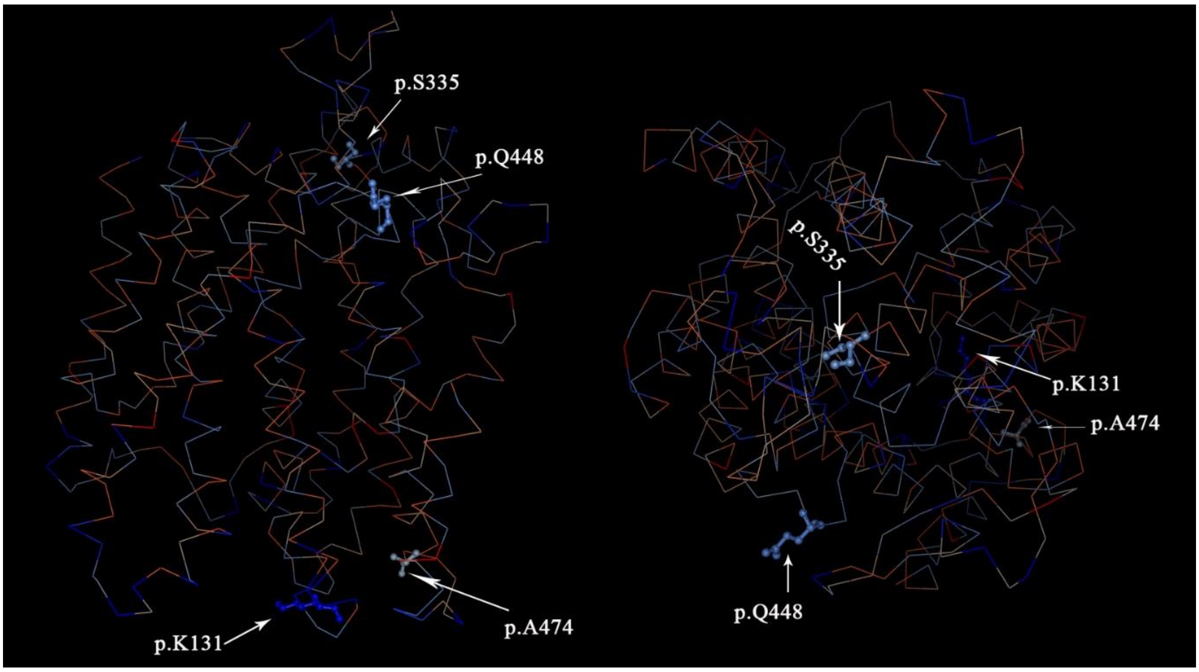

Supplemental Figure 3. The 3D SWISSMODEL of SGLT2 (Residue range: 25 to 497; Based on template: 2xq2A; Sequence identity: 31%; Resolution: 2.73 (X-RAY)) and the positions of the mutations p.K131, p.S335, p.Q448 and p.A474 in it.

Supplemental Table 1. Five missense mutations in SLC5A2 gene identified in this study and their pathogenicity predicted by three different in silico programs (SIFT, PolyPhen-2 and Mutation Taster)

Exon	Nucleotide changes	Amino acid change	SIFT	PolyPhen-2	Mutation Taster
Exon 4	c.393G>C	p.K131N	Damaging	Probably Damaging	Disease causing
Exon 8	c.1003A>G	p.S335G	Damaging	Probably Damaging	Disease causing
Exon 11	c.1343A>G	p.Q448R	Tolerated	Probably Damaging	Disease causing
Exon 11	c.1420G>C	p.A474P	Damaging	Probably Damaging	Disease causing
Exon 13	c.1739G>A	p.G580D	Tolerated	Benign	Polymorphism

Supplemental Table 2. The predicted effects of the deletion c.886(-10_-31)del in intron 7 on the putative branching point motif by in silicon program HSF3.0

cDNA Position	Branch Point motif	CV for reference sequence (0-100)	CV for mutant sequence (0-100)	Variation
c.886-34	CCCGCAA	78.29	49.51	Site broken
c.886-33	CCGCAAG	50.69	70.37	New site
c.886-29	AAGCGGG	16.84	70.36	New site
c.886-21	AGCTGAA	79.62	39.23	Site broken

CV, cut-off value
